# Bringing a humanistic approach to cancer clinical trials

**DOI:** 10.3332/ecancer.2017.738

**Published:** 2017-05-18

**Authors:** Roberto Jun Arai, Elaine Santana Longo, Maria Helena Sponton, Maria Del Pilar Estevez Diz

**Affiliations:** Núcleo de Pesquisa, Instituto do Câncer do Estado de São Paulo, Faculdade de Medicina da Universidade de São Paulo, Hospital das Clínicas da Faculdade de Medicina da Universidade de São Paulo, São Paulo 01246-000, Brazil

**Keywords:** humanization, cancer, clinical trials

## Abstract

In this article, we describe some practical aspects that promote the humanisation of clinical research. Actions are not limited to improving the communication skills of medical staff but also include maintenance of care continuity, accessible written information, and application of theoretic models such as shared decision-making and management of stress in decision-making under uncertainty. We believe that a comprehensive strategy will increase patients’ motivation to participate in and adhere to clinical research.

The application of evidence-based medicine (EBM) in the 21st century as a practice built upon data derived from rigorous study protocols has proven to be fundamental. In order to provide the best level of evidence, the complexity and sample size of clinical trials have increased drastically. In parallel, restrictive selection criteria to enrich the population strongly limited accrual. As a consequence, screening success has decreased and fewer subjects are completing studies [1, 2]. Loss of data may critically affect interpretation of study results, and missing data may be managed using the last observation carried forward method. The Consolidated Standards of Reporting Trials (CONSORT) guidelines recommend intention to treat to improve the quality of randomised clinical trials. This artificial approach appears valid, but nevertheless underestimates treatment effects due to dilution of the effects of noncompliant individuals.

Poor compliance generally refers to deviations in patient behaviour from what was advised by medical staff. Common explanations reported by patients are diverse and include forgetfulness, other priorities, the decision to skip doses, lack of information/communication, and emotional factors [3]. Patient adherence depends on a vast array of variables that influences decision-making. Protocol redundancies and time inefficiency often found in lengthy studies increase the time commitment required of patients and may also promote attrition. Another issue includes the perception of lack of incentives to complete the study; this is the case in control/placebo group patients, who may believe that signs of clinical benefit are no apparent. Compliance, however, should be carefully analysed in palliative oncology trials where dropouts are likely attributable to clinical deterioration [4]. Dropouts are also influenced by comorbidities such as depression and anxiety, and attrition may be promoted if patients with these comorbidities are not treated adequately [5]. Thus, the desire to participate in a clinical trial and willingness to complete the study are multifaceted. It is imperative to address individual education and values from patients’ perspective and expectations from physicians’ perspective *a priori*.

Differing viewpoints that follow the best evidence alone are not always sufficient to ensure the most rational clinical management. The EBM paradigm thus poses limitations in the ability to actively incorporate health-related quality of life [6]. Sharing decisions with patients instead of making decisions on behalf of patients is critical. Studies have consistently noted gained confidence and deeper involvement of patients in their treatments [7]. Shared decision-making (SDM) may be defined as “an approach where clinicians and patients share the best available evidence when faced with the task of making decisions, and where patients are supported to consider options, to achieve informed preferences” [8]. The conceptual principle of SDM deepens patient-centred care [9]. In routine practice, SDM should start with information provision to support decision-making. For the best results, information must be of high quality [9, 10]. The participation in randomised controlled trials (RCTs) can be difficult. Partial preference designs may improve protocol compliance but still have little impact on recruitment [11]. Strategies addressing the awareness of potential participants of the health problem being studied and its effects on their life and that engage potential participants in the trial process are likely to increase recruitment to RCTs [12].

Patients’ individuality strongly influences, if not determines, the decisions made. Value-based medicine addresses individuality and can provide clinical skills to link generalised scientific knowledge of EBM to particular preferences. This concept was also referred to as humanised medicine [13, 6]. In the literal sense, humanisation grants human traits, making benevolent, gentle, compassionate or generous. From a philosophical point of view, humanism aims to understand individuals in society in a moral context and promotes humanitarian values such as solidarity and empathy [14]. The injection of “humanistic” terminology into medical education and practice, however, has been used as to reforming effect. It attempted to recover human values in times when ethics were elusive. Humanistic healthcare highlights human interactions, including patients’ families, friends, and medical staff. The construction of a partnership is wired by open communication that is particularly sensitive to patient expectations and values [15–17]. The humanistic care process may promote a humanistic–altruistic system of values; development of a trusting relationship; acceptance of positive and negative feelings; systematic use of the scientific problem-solving method for decision-making; and promotion of a supportive environment. These are some examples of Watson’s Theory of Human Caring [18].

Primacies of communication, attention to the problem, and empathy build trust over time [19]. Trust mediates positive outcomes, including adherence to treatment and satisfaction [20]. In a study conducted in 710 cancer patients in Germany, empathy seemed to be an important requisite for information provided by physicians, and this pathway led to a preventive effect on depression and improved quality of life [21]. Conversely, physicians’ stress negatively influences these relationships. In a retrospective correlational study in over 20,000 patients with diabetes mellitus, investigators found that patients of physicians with high empathy scores had significantly lower rates of acute metabolic complications [22].

Cancer is a potential life-threatening disease and participation in cancer clinical trials comprises an additional and stressful crossroad [2, 23, 24]. Patient-centred concerns commonly include understanding of assignment to placebo or no treatment, potential adverse effects, and impact on quality of life. Cancer clinical trials also take volunteers on a journey with essentially unknown outcomes. Nevertheless, the majority of the British population consider it important for the National Health Service to offer opportunities for patients to take part in clinical research. This paradox may be supported by the perception that patients are under certain levels of risk and uncertainty but that clinical trials are critical for the development of new treatments [25]. Efficient clarifications that clarify steps to move forward are of great importance in managing choices under uncertainty. Thus, accessible information, individualisation, and care continuity are some important aspects that influence decisions that finally aim a maximum expected practical utility [26].

One of the key documents used as a basis for communication and decision-making is the informed consent form (ICF). Clinical research programmes frequently include multinational collaborations in which documents are translated to local languages. Although a review is recommended to ensure that the translated document is adequate, its understanding raises constant concerns. The majority of the population of most countries, including the USA considers ICF a difficult instrument to understand. In low- and middle-income countries, this scenario is likely to be even worse [27, 28]. A humanistic approach using a comprehensive strategy may be implemented in order to dissipate patients’ sense of ‘gambling with their lives’ when deciding to participate in a cancer trial. Herein, we describe some practical aspects of the humanisation approach in routine settings.

## Improving consent process communication

We developed a standard process for ICF review that includes an easy-to-read approach [28]. The revision uses familiar terms and ideas, short words and texts, and bullet points to break up long explanations. Most of the changes are well accepted by the local Ethics Committee. However, revisions frequently trigger long discussions with study sponsors that are sometimes insensitive to relativisms found worldwide where theories and practices differ by culture. As ICF revision is a responsibility of local investigators per good clinical practice (GCP), normally, reviewed ICFs are eventually accepted, since key information is not typically changed. The face-to-face process for consent was improved by training medical staff to use familiar words to address specific protocol details. Extra time is also offered to patients, who are strongly advised to take the ICF home and to discuss it with family and friends. This step is of particular importance, as participants usually include a family member in the SDM process.

## Intelligent guiding

Once the patient agrees to participate in the trial, a nurse with a clinical research background monitors participants according to the tenets of the primary nursing model and humanistic care. This model is intended to strengthen a cooperative relationship of patients and medical staff. Primary nurses schedule procedures, provide specific protocol-related information in cases of doubt, answer particular questions, and review critical events. Consistent contact also augments patients’ confidence in medical management and promotes better quality of information exchange. Any additional orientation is promptly offered to patients, particularly during protocol execution. The research nurses play a key role as accessible resources of organised and personalised information.

## Maintenance of care continuity

Patients believe that good communication concentrating predominantly on their relationships with individual healthcare providers is key to improving overall continuity of care [29]. Cancer care is usually fragmented among different providers (radiologists, oncologists, and surgeons) in a myriad of scenarios that include specialised treatments, outpatients, inpatient conditions and management of comorbidities. Participation in clinical research may comprise another obstacle towards care continuity, making patients feel increasingly disconnected with their primarily care provider [30]. Moreover, modern trials often include a referral network involving several institutions and specialised organisations to speed up accrual. The level of candidates’ interest in study referral is influenced by several variables, including age, treatment satisfaction, and clinical condition. Quantitative survey results suggested that cancer patients who lack interest in clinical trials may be driven to participate by their desire to remain under the care of their personal oncologist [31]. The decision to participate in a trial is also influenced by protocol-specific elements (e.g., burdensome study procedures, number of visits, medication washout). Thus, any investigators involved would require a profound understanding of personal values and expectations of each candidate, which is not always possible given limited patient contact. External referrals are therefore applicable only in select cases [32], and internal referrals within the same medical specialty should be controlled or even restricted. Physicians with no experience of the host institution, particularly clinical oncologists, are encouraged to practice and participate in a clinical research programme as coinvestigators under supervision. Experienced investigators may manage their own patients rather than referring to specialised clinical trialists.

## Specific training

The humanistic care policy is continuously emphasised by the institution to medical and administrative staff as a pillar for cancer patient care. A variety of training to introduce humanistic care competencies into clinical research activities are constantly offered and monitored as to update and upgrade skills throughout a physician’s career. Medical research staff are trained in research standards such as GCP and its interpretation from the perspective of patient values. Seminars on scientific methodology in clinical research intersect with those related to the humanities. For instance, discussions about sample size are connected with patient social characteristics that may influence study compliance. Economic particularities and local relativisms of the study population are common subjects considered in eligibility criteria. Assertive communication to assess individual values during medical visits is constantly supervised by senior physicians as part of the on-job-training method to enhance insight into the lived experiences of students, residents, and patients.

Improvements regarding communication are evaluated by surveying patients with a questionnaire that examines their level of understanding of their rights, study procedures, and ICF content. This monitoring is a component of the individual tracer activity implemented in the institution, using actual patients as the framework for assessing compliance with standards. Individual tracers follow a patient through his or her course of care.

￼Several aspects of humanised clinical research appear to be challenging (Table 1), as study protocols are conducted according to strict standardised procedures to ensure data are trustworthy. The shift in EBM has been viewed as colliding with the principles of humanistic care. However, coalescence is an opportunity to simultaneously address the objectives of patients, physicians, and research promoters. Patients’ expectations may be better achieved when options are well evaluated. Although cancer trial participants commonly report that altruism contributed to their decision to enrol, it is rare for this to be the primary motivation for study participation. Indeed, the decision to participate frequently stems (at least partially) from the possibility of direct benefit. In early-phase trials and among patients with poor prognoses, altruism is least often the motivator [33]. Study participation may be compromised if the trial’s objectives are not completely understood. Patient adherence may be poor if potential ‘real-word’ effects such as side effects or lack of efficacy are not well described. The failure of candidates to acknowledge the protocol design may culminate in a disastrous level of participation. In our institution, after introducing the humanistic approach to clinical research, we observed a lower percentage of dropouts in comparison with other reports, in which attrition rates ranged from 20% to 30% [34]. The humanistic approach to clinical research activities took several years to implement due to the time required to develop training, validate standard procedures, and instil a humanistic culture. The institution and specifically the research centre are evaluated by patient satisfaction, dropouts, and compliance. Influences in attrition rates, however, are diverse, including the abundance of single-arm and open label trials found in cancer research; these are due to improved access to life-threatening diseases embraced by the US Food and Drug Administration [35]. In these types of studies, there is no uncertainty regarding which patients will receive the placebo, and they are likely to be well accepted by patients.

## Conclusion

In this article, we have described some practical aspects that promote the humanisation of clinical research. Actions are not limited to improving the communication skills of medical staff but also include the promotion of care continuity, accessible written information, and application of theoretic models such as SDM and management of stress in decision-making under uncertainty. We believe that a comprehensive strategy will increase patients’ motivation to participate in and adhere to clinical research. The supportive environment provided through humanisation improves communication quality and ultimately transforms the relationship between investigator and volunteer to one of trust.

## Figures and Tables

**Table 1.** Humanistic approach to clinical research.

Actions	Results	Pitfalls
Improving consent process communication	Accessible written information provided through easy-to-read approach. Improved consent process promotes shared decision making.	Informed consent form extensive reviews may not be accepted by study promoters. Humanistic approach may cause delays that are not always acceptable.
Intelligent guiding	Patients feel confident and well monitored. Adherence and quality of data are improved.	Staff training is longer and costly. This model requires a larger professional-to-patient ratio.
Maintenance of care continuity	Improve patient-centred care. Incorporating clinical research activities by physicians bring additional expertise including international standards in clinical data management and access to new interventions.	Increased number of clinical research-related trainings is time consuming. Medical research may be interesting only to selected physicians.
Specific training	Cultivation of values of empathy, justice, and compassion in a very delicate clinical research environment.	Instillation of humanistic culture in clinical research is longer and may take several years.
